# Using the Angoff method to set a standard on mock exams for the Korean Nursing Licensing Examination

**DOI:** 10.3352/jeehp.2020.17.14

**Published:** 2020-04-22

**Authors:** Mi Kyoung Yim, Sujin Shin

**Affiliations:** 1 Korea Health Personnel Licensing Examination Institute, Seoul, Korea; 2 College of Nursing, Ewha Womans University, Seoul, Korea; Hallym University, Korea

**Keywords:** Angoff method, Nurses, Nursing licensure, Standard setting, Republic of Korea

## Abstract

**Purpose:**

This study explored the possibility of using the Angoff method, in which panel experts determine the cut score of an exam, for the Korean Nursing Licensing Examination (KNLE). Two mock exams for the KNLE were analyzed. The Angoff standard setting procedure was conducted and the results were analyzed. We also aimed to examine the procedural validity of applying the Angoff method in this context.

**Methods:**

For both mock exams, we set a pass-fail cut score using the Angoff method. The standard setting panel consisted of 16 nursing professors. After the Angoff procedure, the procedural validity of establishing the standard was evaluated by investigating the responses of the standard setters.

**Results:**

The descriptions of the minimally competent person for the KNLE were presented at the levels of general and subject performance. The cut scores of first and second mock exams were 74.4 and 76.8, respectively. These were higher than the traditional cut score (60% of the total score of the KNLE). The panel survey showed very positive responses, with scores higher than 4 out of 5 points on a Likert scale.

**Conclusion:**

The scores calculated for both mock tests were similar, and were much higher than the existing cut scores. In the second simulation, the standard deviation of the Angoff rating was lower than in the first simulation. According to the survey results, procedural validity was acceptable, as shown by a high level of confidence. The results show that determining cut scores by an expert panel is an applicable method.

## Introduction

### Background/rationale

In the criterion referenced evaluation, such as licensing tests, it is important to establish criteria for acceptance and rejection. In Korea, the written nurse licensing test still uses a uniform criterion of more than 60% of the total score [1]. In other countries, however, it is common to set acceptability criteria by establishing standards. The National Council of State Boards of Nursing (NCSBN) is a nonprofit organization that sponsors the US National Council Licensure Examination (NCLEX), which organizes specialized tests for registered nurses and licensed practical nurses. The NCSBN operates several committees for the development and implementation of these national examinations for nurses. There is also a panel of judges, along with the item development committee, item writers, and item reviewers. The item review team consists of qualified item writers, item reviewers, nursing professors, and new graduate nurses. Their most important role is to determine the NCLEX cut score, and pilot tests are conducted to review the results and confirm the feasibility based on a review of any possible problems [2]. According to the NCSBN, because nursing practice changes over time, it is necessary to reconsider the acceptability criteria over time. In accordance with the agreement made at the meeting of NCSBN representatives in 1989, the Board of Directors assesses the suitability of the passing standard every 3 years or whenever the test plan is changed. This means that the test plan and acceptability criteria will be reset every 3 years [3].

If international graduates want to work as a nurse in the United Kingdom, they must pass the Nurse Competency Examination (a test of competence). This test is composed of a 1-step computer-based test (CBT) and the second step is a practical test (an objective structured clinical examination). The CBT exam has 120 items, of which a score of 66% is needed to pass. This passing criterion is predetermined by the expert panel; although it is currently 66%, this threshold is not inherently fixed. The Nursing and Midwifery Council periodically checks this criterion, taking into account the difficulty of test items and the level of candidates [4].

A number of studies have pointed out problems with the Korean Nursing Licensing Examination (KNLE) compared to the exams of other nations [3,5,6]. However, institutional change has not yet been achieved because no rational standards have been established.

Various standard setting methods have already been proposed, of which criterion-referenced and test-centered methods are suitable for written tests that consist of multiple choice questions [7]. The Angoff method, which was proposed by Angoff [8] in 1971, is the most widely used method. In this framework, content experts examine each test item and estimate the probability that a minimally competent person will correctly answer the item on the test [8]. The Angoff method is widely applied to licensing or achievement tests, is easy to understand since it is much simpler than other methods, and has been deemed to be the method that best balances between technical suitability and practicality [9].

### Objectives

The purpose of this study was to establish criteria for mock KNLE exams using the Angoff method and to analyze the results. In the Angoff procedure, the expert panel discussed the minimum competency of a licensed nurse and rated each item of the exam to determine a cut score for the whole exam based on the level of a minimally competent person. The process was conducted for 2 mock exams and the scores were compared with the existing national examination passing score. The specific research goals were as follows: (1) to discuss the minimum competency level for the KNLE; (2) to set the cut score of the mock exams for the KNLE; and (3) to examine the procedural validity of establishing the criteria by investigating the responses of the standard setting panelists.

## Methods

### Ethics statement

All the study participants indicated that they understood the purpose of the study and agreed to participate voluntarily. When informed consent was obtained from the participants, it was explained that the participants’ anonymity would be preserved, that the results of the evaluation and questionnaire would used only as research data, and that any personally identifiable data would be discarded after the study is finished.

### Study design

It is the analysis of the panel discussion for the standard setting of the mock exam.

### Participants (standard setting panelists)

The number of standard setting panelists is recommended to be around 10 to 15 for each subject, but it depends on the composition of the exam or institutional circumstances [10]. The group of experts on the standard setting panel for this study consisted of 16 nursing professors. The experts actively taught students at nursing colleges and also worked as item developers or committee members of the national licensing exam. Since the test subjects were drawn from 8 major subject areas, at least 1 person for each of the 8 specializations was included. In accordance with the advice of a nursing expert, the panel was grouped into similar subjects, and the number of standard setters for each subject was increased by arranging 2 specializations in a single group for cross-checking. The composition of the standard setting panel and the composition of each subject are listed in Table 1. We sought to secure at least 2 panelists per subject and to double the number of adult nursing panelists considering the number of questions. The final panel consisted of 5 specialists in adult nursing, 2 in fundamental nursing, 2 in maternity nursing, 1 in pediatric nursing, 1 in community nursing, 1 in psychiatric nursing, 3 in nursing management, and 1 in medical health legislation.

### Setting

#### Mock examination

The KNLE consists of 295 items distributed across 8 subjects. In 2018, the distribution of each subject on the KNLE and the acceptability criteria, item number, and scores are as shown in Table 2. The number of examinees of the 58th KNLE, administered on January 2018, was 20,731 and the pass rate was 96.1%. In the last 5 years, the lowest pass rate was 93.8% (2016) and the highest pass rate was 96.7% (2014 and 2015). It is relatively stable, but differences of approximately 3%–4% appear from year to year [11]. Since the KNLE is not open to the public, this study analyzed 2 mock exams that are used to prepare for the national examination of nurses [12]. Although these mock examinations were not official (as they were published by third-party companies), they were expected to have similar properties to the KNLE, as they had the same subject distribution, item format, and number of items.

#### Implementation of the Angoff method

The standard setting procedure was conducted in the following order: pre-education, discussion of minimum competency, evaluation, result confirmation and discussion, correction, and final result. The workshop was conducted for 2 days, and the schedule is presented in Table 3. In the pre-education, the purpose of the study was introduced, followed by the theoretical background of standard setting, an introduction of prior studies on the minimum competency of new graduate nurses, a presentation on prior studies on standard setting, and a discussion of the Angoff rating method. The panel adjusted the result of the individual ratings after a group discussion, and then made the second adjustment after the entire discussion. The second adjusted score was confirmed by all members and accepted as the final result. The same procedure was repeated for the 2 mock exams.

#### Description of performance levels

The definition of the minimum competency and the level of minimum competency should be discussed to determine the standard. With reference to prior studies on the minimum competency of nursing that were mentioned in the pre-education session, the panelists discussed the minimum competency based on their knowledge and experience [6]. In order to establish an awareness of the level of new nurses at the entry level, the panelists classified the tasks that new graduate nurses can and cannot do for each subject, and provided feedback and corrections through small-group discussions and discussions among the entire panel. We asked the panel to describe in as much detail as possible what they could do and what they could not do at the level of an advanced beginner. Based on the results of each group, a performance level description (PLD) was established to reflect the minimum competency level of new nurses after the discussion.

#### Angoff rating

Panelists were assigned items for each subject, and the Angoff rating was conducted individually. The panelists were asked to judge the probability that the minimally competent person would answer correctly by assigning each item a number between 0 and 100. For example, a score of 80 would mean that the probability of the minimally competent person answering the item correctly is 80%. In other words, if 100 minimally competent examinees replied as a group, 80 of them would answer correctly. We cautioned the panel members to assume the probability of correctly answering for the minimally competent person, rather than the average level of the competent test taker. The rating results were collected and discussed as a group, and the results were adjusted. The second adjusted score was confirmed by all members and accepted as the final result.

Each team was evaluated by panel members from 2 specializations. Seven panelists in adult nursing and fundamental nursing evaluated 70 items in adult nursing and 30 items in fundamental nursing, for a total of 100 items. Three panelists in maternity nursing and pediatric nursing evaluated 70 items, 2 panelists in community nursing and psychiatric nursing evaluated 70 items, and 4 panelists in nursing management and legislation evaluated 55 items.

### Survey for procedural validity

Setting standards is a decision-making process [7]. Therefore, the validity of the results refers to how well the procedure was followed, whether the panel was properly configured, and whether the procedure for setting the criteria was closely followed, and the degree of confidence in the calculated reference scores is the basis for verifying the validity of the procedure. Therefore, after the cut score was set, the panel members completed a recognition survey that evaluated the understanding of pre-education, the appropriateness of the procedure, and the appropriateness of the results on a 5-point scale.

### Statistical analysis

Descriptive statistics were applied for the results of the panel discussion and the survey results.

## Results

### Performance level description for defining minimum competency

Based on the content of this study, the minimum level of competence of new graduate nurses derived from group discussions is shown in Table 4. In particular, a significant finding is that it was possible to better understand the achievement level of each subject by deriving the PLD for each subject.

### Cut score

Table 5 shows the results of setting the acceptability criterion by applying the Angoff method (Dataset 1). For the first mock exam, the cut score was 74.4 on a 100-point scale, and for the second mock exam, it was 76.8 points. When the measurement error (standard error, SE) was calculated, the measurement error of the first mock exam was found to be 2.2, and the measurement error of the second mock exam was 1.6. Therefore, the passing score with the measurement error applied ranged from a minimum of 72.3 to a maximum of 76.6 for the first mock exam, and from a minimum of 75.3 to a maximum of 78.4 on the second mock exam. Considering the first and second error ranges, if the average passing score for the 2 exams is used, the appropriate passing score would be between 75 and 76 points. The overall score is the sum of the ratings of each subject, so the reference score for each subject can be presented as shown in Table 6.

### Survey results

Table 7 shows the frequency of responses to 13 questions on the procedure for setting the cut score, and the average score and the standard deviation (SD) were scored on a scale of 5 points. The higher the score, the more positive the reaction (Dataset 2.).

The average level of understanding of the pre-education was 4.56 points, and the response frequency of ‘agree’ and ‘strongly agree’ was 93.75%. Through pre-education, the purpose of setting the cut score was well understood, and the panel clearly recognized the task that was to be done. The average score for whether the definition of the minimum competency was clear was 4.69 points, with response frequencies of ‘agree’ and ‘strongly agree’ of 31.25% and 68.75%, respectively. In a previous study of a similar process for medical doctors, the panel responded that they had difficulties defining the minimum competency, and that the definition of the minimum competency did not help greatly in the evaluation [13]. However, in this study, a consensus on the minimum competency was established smoothly. Regarding the usefulness of the PLD, only 6.25% responded ‘disagree,’ while 93.75% responded ‘agree’ or ‘strongly agree.’

The panel also reported no difficulties in assuming the probability that respondents with the minimum competency would answer questions correctly. No respondents felt that they had difficulties when responding to the item, “It was easy to assume the response probability of the minimum competent person.” The definition of the minimum competency and the assumption of response probability are very important parts of the Angoff standard setting method as ways to increase the procedural validity of this method. The panelists agreed that the discussion was very meaningful, that the information and time provided for the discussion were adequate, and that the discussion was smooth. The average score for confidence in the first cut score was 4.44, while the confidence score for the cut score generated by the entire panel was higher (on average, 4.56).

Participants were asked about the advantages and disadvantages of applying this deliberation method to the national licensing examination, and the reasons were described. All 16 panelists agreed on the applicability of this method. The panelists agreed that they were able to determine the cut score of the exam according to the difficulty of the items, and endorsed the validity of this deliberation method, which can determine the acceptability criteria based on the content and a cut score according to difficulty.

## Discussion

### Key results

This study derived the score for the acceptability criteria by applying the Angoff method to mock exams for the national examination of nurses, and the results show that the application of this method of determining the cut score by an expert panel can realistically produce stable results.

In terms of the method for setting the criteria, the application procedure of the Angoff method was evaluated as having been properly applied, and its potential for application is expected to be positive. The discussion on the minimum competency was informative, the panel composition by specialization was appropriate, and the method of organizing the evaluation and group discussion by classifying similar subjects into the same group increased the efficiency of the panel operation. It seems that the members were satisfied with the implementation of the technique.

### Interpretation

A noteworthy change in the 2 simulation ratings calculated by applying the Angoff method was that the variance in the panel ratings was lower in the second trial. According to Table 5, the SD of the first mock exam was greater than the SD of the second mock exam in both rounds. Thirty-six items had an SD of more than 20 points based on the first round of the first mock exam discussion, but only 3 items had such a large SD after the first rating for the second mock exam. Through the evaluation, discussion, and coordination process, the panelists were found to have a similar level of awareness of the level and difficulty of the target test and reported that it had a learning effect. The importance of education and experience was seen.

Because there may be a difference in the difficulty between the simulated tests and the actual national test, the interpretation of the score is limited. The KNLE has a high pass rate of 96% to 97% on average based on a cut score of 60% of the total score. For the mock exams, the panel judged that a cut score based on minimum competency would result in a passing score of 75–76 points out of 100 points. In previous studies of national tests of medical doctors, medical recorders, and radiologists, the cut scores derived by applying the Angoff method were all higher than the existing reference scores of 60 out of 100. When the modified Angoff method was applied to the 74th national test of the Korean medical licensing exam, the reference score was 61.4, and when the modified Angoff method was applied to the 81st national test, the reference score was 60.93 points and cut scores of 72.36 and 73.01 points were derived under 3 different conditions [13,14]. When the modified Angoff method was applied to the national examination of medical recorders and radiologists, cut scores of 62.95 points for medical recorders and 71.27 points were obtained for radiologists [14,15].

Compared with the results of these previous studies, a similarity is that the passing score of the nurse national exam simulation tests was higher than the existing reference score, but there are limitations in generalizing this finding to the nurse national exam because the difficulty of mock exams is not exactly the same as that of the KNLE .

Furthermore, distributing the items across each subject was appropriate as a way to increase the efficiency of setting standards. Ferdous and Plake [16] in 2005 set the standard for the K-12 academic achievement assessment in the United States and, when evaluating all items, made the evaluators assess a partial subset of items in consideration of the fatigue of the evaluators, with a resulting decrease in reliability. It was reported that only 50% of the items were evaluated to be the same as the overall results. Buckendahl et al. [17] in 2010 studied the application of the Angoff method for a partial set of items from the Canadian dental licensing test. In previous studies, the panel members reported that it was burdensome to evaluate items outside of one’s specialization [13,14]. Dividing the subjects into sub-specializations, such as on the KNLE, and then evaluating and combining each subject better reflected the panel’s expertise. This method is suitable for effective evaluations.

The panel members who participated in the national exam for nurses as item developers had a very positive response to the application of the expert rating method (i.e., the Angoff method), which was similar to the results of previous studies of medical doctors, medical recorders, and radiologists [13,14].

According to the panel awareness survey, the participants were strongly in agreement with the need to improve the current system for determining the cut score. The panelists recognized that the discussion on the minimum performance ability and minimum performance ability of the nurse’s license test was very helpful and necessary. As qualifications for the panelists, cut score deliberation experience was identified as important, in addition to questionnaire presentation experience, educational experience, and clinical experience. The participants expressed their hope to have further opportunities for educational workshops and experiences like this study.

### Conclusion

Therefore, based on the results of the study, the specific points that should be considered when applying the pass-screening method to the national nurse test are as follows.

First, it is necessary to prepare a formal minimum competency level description (PLD) that can be applied immediately in deliberations on the passing score through an in-depth consideration of the definition and level of the minimum competency. A meaningful description of the achievement level was derived through this study, but it is recommended to produce a more rigorous description by formally gathering opinions through venues such as conferences and research associations in order to draw more common opinions from more stakeholders.

Second, with the current composition of subjects on the nurse national examination, the evaluation of each subject is valid, and 4 to 5 evaluators are recommended for each subject. It is recommended that the panel should have minimum qualifications, including at least 5 years in item development experience, education experience, and practical experience. In addition, it is necessary for the National Assembly to secure a pool of experienced personnel by expanding workshops and training opportunities for deliberation on cut scores.

Third, in terms of measurements, it is proposed to establish criteria for each subject with due consideration of measurement error, with the goal of determining a final score that is within ±1 SE of the average score of the panel.

If education and training are continued, it is highly probable that the Angoff method will be applied to the KNLE. Nonetheless, even for a consistent procedure, the adaptability, readiness, and acceptability will differ depending on the profession. This study confirmed that nursing professors reported a high adaptability and acceptability of the application of alternative cut scores.

## Figures and Tables

**Table 1.** General characteristics of the standard setting panelists (N=16)

Characteristic	Category	No. (%)
Gender	Female	16 (100.0)
	Male	0
Age (yr)	30s	2 (12.50)
	40s	5 (31.25)
	50s	8 (50.00)
	60s	1 (6.25)
Experience as item developer	Less than 3 years	4 (25.00)
	3–5 years	3 (18.75)
	5 years or more	9 (56.25)
Experience as educator	Less than 10 years	2 (12.50)
	10–15 years	4 (25.00)
	15 years or more	10 (62.50)
Specializations	Adult nursing	5 (31.25)
	Fundamental nursing	2 (12.50)
	Maternal nursing	2 (12.50)
	Pediatric nursing	1 (6.25)
	Community nursing	1 (6.25)
	Psychiatric nursing	1 (6.25)
	Nursing management	3 (18.75)
	Medical health legislation	1 (6.25)
Team for ratings	Team 1 AN-FN	7 (43.75)
	Team 2 MN-Ped	3 (18.75)
	Team 3 CN-Psy	2 (12.50)
	Team 4 NM-MH	4 (25.00)

AN, adult nursing; FN, fundamental nursing; MN, maternity nursing; Ped, pediatric nursing; CN, community nursing; Psy, psychiatric nursing; NM, nursing management; MH, medical health legislation.

**Table 2.** Subjects, number of items, and cut scores for passing

No.	Subject	No. of items	Score	Standard for passing	
				Subjectcut score	Total cut score
1	Adult nursing	70	70	28	
2	Maternal nursing	35	35	14	
3	Pediatric nursing	35	35	14	
4	Community nursing	35	35	14	
5	Psychiatric nursing	35	35	14	
6	Nursing management	35	35	14	
7	Fundamental nursing	30	30	12	
8	Medical health legislation	20	20	8	
Total		295	295		177

**Table 3.** Schedule of the Angoff standard setting procedure

Day	Time	Procedure
Day 1	10:00–10:30	Registration
	10:30–11:00	Introduction and confirm of participants
	11:00–12:00	Pre-education
	12:00–13:00	Lunch
	13:00–14:30	MCP discussion
	14:30–16:30	Mock exam 1: Angoff rating
	16:30–17:00	Group discussion, first
	17:00–18:00	Group discussion, second
	18:00–19:00	Dinner
	19:00–20:00	Panel discussion and decision on the final score
Day 2	09:00–10:00	Orientation
	10:00–11:30	Mock exam 2: Angoff rating
	11:30–12:00	Group discussion, first
	12:00–13:00	Lunch
	13:00–13:30	Group discussion, second
	13:30–14:00	Panel discussion and decision on the final score
	14:00–15:00	Survey

**Table 4.** Performance level description of the overall exam and each subject

General	General PLD
General description	1. Understand changing health care settings and policies, comply with ethics and laws, distinguish between normal and abnormal life cycles of subjects, perform nursing for health promotion, patient monitoring, administering medication, pre- and post-test care, perioperative care, nursing for medical treatment, and nursing care for discharge.
	2. Understand the management system of nursing units, cope with emergencies and determine priorities, and perform limited monitoring of high-risk subjects' health and special testing and treatment.
Subject	Subject PLD
Adult nursing & fundamental nursing	1. Health monitoring for common diseases, administering medication, pre- and post-test care, perioperative care, nursing for medical treatment, and nursing care for discharge can be performed.
	2. The nurse can partially monitor the condition of high-risk subjects in the intensive care unit or operating room, or care for special examinations and treatments.
Maternal nursing	1. Perform normal pregnancy, delivery, and postpartum nursing care.
	2. Identify high-risk pregnancy, delivery, and postpartum nursing care problems.
	3. Perform nursing before and after a cesarean section.
	4. Perform nursing before and after gynecological surgery.
	5. Identify genital health problems.
	6. Distinguish between normal and abnormal life cycle health problems.
Pediatric nursing	1. Understand and identify the characteristics of normal children at different stages of development (newborn, infancy, toddler, preschool age, school age, and adolescence), and plan and carry out nursing activities necessary to maintain and promote good health.
	2. Understand the concept of child and family nursing and apply basic principles.
	3. Differentiate between normal and abnormal stages of development of the child.
	4. Assess children with health problems at different stages of development and systems, understand the nature of health problems, and understand key interventions.
	5. Plan and carry out nursing interventions for children with health problems.
	6. Perform community resource linkages.
Psychiatric nursing	1. Understand the concepts of mental health and mental illness.
	2. Distinguish between therapeutic and non-therapeutic communication.
	3. Understand the need for nursing intervention techniques.
	4. Distinguish between sensory perception and thinking disorder (dyslogia) and develop a nursing plan.
Community nursing	1. Understand national and international health care policies and planning health projects.
	2. Use public health, health care, and welfare services resources and work with health care teams.
	3. Provide health education through professional capacity building.
	4. Manage infection control and accident prevention.
Nursing management	1. Understand nursing history.
	2. Apply ethical decision-making processes in clinical settings.
	3. Understand the need for a positive nursing professional identity.
	4. Have knowledge and skills in planning, organizing, personnel, commanding, and controlling functions for nursing care.
	5. Apply the acquired knowledge, skills, and attitudes to problem solving for nursing unit management.
Medical health legislation	1. Understand the health and medical laws and regulations that change with the health care environment.
	2. Have a basic understanding of the statutes that must be followed in working as a nurse in clinical settings.
	3. Have a basic understanding of the statutes that must be followed in working as a nurse in community settings.

PLD, performance level description.

**Table 5.** Cut score of each round and final cut score

Test	First round		Second round		Final cut score	
	Mean	SD	Mean	SD	Mean	SE
Mock exam 1	74.2	8.8	74.4	8.6	74.4	2.15
Mock exam 2	76.8	6.2	76.8	6.2	76.8	1.55

SD, standard deviation; SE, standard error.

**Table 6.** Cut scores for each subject and total score

Subject	No. of items	Mock exam 1			Mock exam 2		
		Mean^a)^	SD	SE	Mean^a)^	SD	SE
Adult nursing	70	69.8	8.8	3.3	78.3	6.8	2.6
Fundamental nursing	30	79	4.6	1.7	79.5	4.4	1.7
Maternal nursing	35	80	8.1	4.7	73	5.6	3.2
Pediatric nursing	35	78.2	6.8	3.9	76.6	6.3	3.6
Community nursing	35	68.1	7.5	5.3	73.6	6.4	4.5
Psychiatric nursing	35	70.2	8.3	5.9	76.7	5.6	4
Nursing management	35	78.5	4.4	2.2	78.8	4.9	2.5
Medical health legislation	20	78.5	5.7	2.9	76.8	5.1	2.6
Total	295	74.4	8.6	2.2	76.8	6.2	1.6

SD, standard deviation; SE, standard error.

a) Mean transformed into a score out of 100.

**Table 7.** Survey results of panelists

No.	Question	Frequency (%)					Score	
		Strongly disagree	Disagree	Neutral	Agree	Strongly agree	Mean	SD
1	Pre-education: understanding and clarity of the research object	0	0	1 (6.25)	5 (31.25)	10 (62.5)	4.56	0.61
2	Pre-education: clarity of the task	0	0	0	4 (25)	12 (75)	4.75	0.43
3	Clarity of the definition of a minimally competent person	0	0	0	5 (31.25)	11 (68.75)	4.69	0.46
4	Ease of assumption of the probability of the minimally competent person responding correctly	0	0	2 (12.5)	7 (43.75)	7 (43.75)	4.31	0.68
5	Usefulness of the performance level description	0	1 (6.25)	0	9 (56.25)	6 (37.5)	4.25	0.75
6	Ease of rating according to guidelines	0	0	0	7 (43.75)	9 (56.25)	4.56	0.50
7	Usefulness of the discussion after individual ratings	0	0	0	1 (6.25)	15 (93.75)	4.94	0.24
8	Usefulness of the group discussion	0	0	0	1 (6.25)	15 (93.75)	4.94	0.24
9	Appropriateness of the information for helping the discussion	0	0	0	2 (12.5)	14 (87.5)	4.88	0.33
10	Enough time to discuss	0	0	0	5 (31.25)	11 (68.75)	4.69	0.46
11	Enough opportunity for participants to discuss	0	0	1 (6.25)	0	15 (93.75)	4.88	0.48
12	Confidence in my cut score	0	0	1 (6.25)	7 (43.75)	8 (50)	4.44	0.61
13	Confidence in the final cut score of the panel	0	0	1 (6.25)	5 (31.25)	10 (62.5)	4.56	0.61

SD, standard deviation.

## Footnotes

**Data availability** Data files are available from Harvard Dataverse: https://doi.org/10.7910/DVN/KSCE32 Dataset 1. Raw data of the cut score setting by panelists for the 1st mock examination. Dataset 2. Response data of the survey.

## Supplementary materials

Data files are available from Harvard Dataverse: https://doi.org/10.7910/DVN/KSCE32

Supplement 1. The audio recording of the abstract.
